# On the Existence and Uniqueness of the Scientific Method

**DOI:** 10.1007/s13752-014-0166-y

**Published:** 2014-04-02

**Authors:** Jorge Wagensberg

**Affiliations:** Faculty of Physics, University of Barcelona, Barcelona, Spain

**Keywords:** Comprehension, Observation, Reality, Research and educational programs, Scientific method

## Abstract

The ultimate utility of science is widely agreed upon: the comprehension of reality. But there is much controversy about what scientific understanding actually means, and how we should proceed in order to gain new scientific understanding. Is there a method for acquiring new scientific knowledge? Is this method unique and universal? There has been no shortage of proposals, but neither has there been a shortage of skeptics about these proposals. This article proffers for discussion a potential scientific method that aspires to be unique and universal and is rooted in the recent and ancient history of scientific thinking. Curiously, conclusions can be inferred from this scientific method that also concern education and the transmission of science to others.

## Three Concepts, Three Hypotheses, Three Principles and Three Benefits

A possible agreement on a single scientific method (SM) would be of high interest both to theory and practice. It would be especially useful to theory because a single, universal SM would make it possible to agree on a definition of science: science is that knowledge arrived at using the SM. The most widely held view on this issue today revolves around the belief that there is no SM that has endured unchanged throughout history and in every discipline. The history of the philosophy of science has amassed various methodologies, each one suited ideally for a particular purpose, and each with its advantages and drawbacks depending on the type of reality being examined with a view to understanding it. Moreover, a single, universal SM would also be useful for creating new scientific knowledge as well as for assessing or criticizing the scientific knowledge that holds sway today. Universality, coherence, and uniqueness are intuitions to be demanded of a possible SM.

The understanding of reality is an accomplishment of the mind that depends on the interaction between the subject and the object of knowledge, that is to say, on observation. Consequently, the most basic conceptual schema revolves around these three concepts: (1) reality, (2) observation (of that reality), and (3) understanding (of that observation of that reality). “The Three Fundamental Concepts (a Conceptual Schema)” section defines these three concepts, and analyzes the relationships between them. “The Three Fundamental Hypotheses (the Limits of Scientific Understanding)” section outlines three fundamental hypotheses, one for each concept. These hypotheses establish the first limits of scientific knowledge, that (1) reality is observable, (2) observation is understandable, and (3) understanding is falsifiable. Reality can only be understood scientifically within the limits set by these three hypotheses. It is impossible to do the science of a reality that cannot be observed either directly or indirectly. Nor is it possible to do science based on an unintelligible observation, even though the reality is observable. And neither is it possible to do science based on an understandable observation if it turns out that it is not falsifiable, even though the reality is observable and the observation understandable.

These three fundamental concepts, established by their respective hypotheses, create the conditions for proposing an SM based on three fundamental principles. “The Three Fundamental Principles (Scientific Method)” section details these principles, one for each concept and its corresponding hypothesis. The most characteristic and novel aspect of this proposal is that these principles are not dictates that compel scientists to use a particular methodology but instead set a trend to be followed. In other words, each of the principles of the SM transpires with a degree of compliance that must be as high as possible in each instance. The more complex the slice of reality to be understood scientifically, the more difficult it will be to observe, and the weaker the application of the SM. For example, SM will go further in understanding the trajectory of a billiard ball once hit than it will in understanding the behavior of a family group of gorillas. However, the two sorts of knowledge will be equally scientific in both cases if the SM is taken as far as it can possibly go. Plainly, accepting the SM will require certain sacrifices on the part of the mind creating knowledge (which an artist, for example, does not necessarily have to accept), but in exchange the knowledge acquired will exhibit certain interesting features. As will be shown in “The Three Fundamental Benefits of the Scientific Method (the Nature of Scientific Comprehension)” section, there is a clear epistemological benefit for each fundamental principle of the method, namely (1) universality, (2) anticipatability, and (3) progress.

The final section, “The Three Intellectual Joys (Psychology of the Acquisition of New Scientific Knowledge: Research and Education),” discusses three psychological effects of scientific comprehension. Curiously, a type of intellectual joy occurs associated with each of the fundamental principles of the schema (and hence associated with each of the fundamental concepts, and each of the hypotheses and fundamental benefits of the method). These are: (1) intellectual joy through stimulus, (2) intellectual joy through conversation, and (3) intellectual joy through understanding.

The most attractive aspect of this conclusion is its direct connection with the principles of the method. In other words, the SM is useful not only for developing science but also for passing it on to others. Again: the SM is relevant to *new* scientific knowledge, whether “new” refers to a single mind (education) or to any other mind (research).

## Three Fundamental Concepts (A Conceptual Schema)

Let us call “knowledge” every mental representation of reality that can be transferred from one mind to another. This perhaps is the difference between knowledge and thinking. A thought can occur inside a mind without a specific expression in a specific language, and it may very well never leave the mind in which it occurred. A piece of knowledge, in contrast, is supported and carried by a slice of reality, since it is reality that it must traverse in order to reach any other mind. Consequently, all knowledge is necessarily finite. A piece of knowledge has weight; it has a size that can be measured in numbers of symbols or packets of symbols. In particular, a thought that cannot be transferred (cannot leap from one mind to another) does not attain the status of knowledge. Any piece of knowledge carrier begins and ends, it occupies a space, be it a written text, a musical score, a painting, a sculpture, or a scientific theory.

I commented earlier that the central intuition that the SM we seek must fulfill is its usefulness for understanding reality. But what purpose does understanding reality serve? There is one utility that is plain to see in history—to survive. This is the crucial point of connection between two concepts of key importance: *natural* selection and *cultural* selection. This idea leads us to two others. In effect, an understanding of reality that anticipates uncertainty ought to enjoy two types of universality. One of these is *internal independence*: the understanding should be as independent as possible of the mind that devised it; in other words, as little preconceived ideology should be invested in the process as possible. The second is *external independence*: understanding should be as independent as possible of the particular slice of reality that we want to understand, and also of the time and place in which this understanding arises. In addition, the comprehension of reality always involves observation, and the manner in which observing is done may suffer from limitations that change with time and place. This means that if observation changes (improves), understanding is also liable to change (improvement). This is the third intuition: the ability of science to progress. So here we have the three first intuitions that the SM we seek must meet: universality, the capacity for anticipation, and the capacity for progress.

These three first intuitions require the mind to be capable of perceiving and recording reality and the changes it undergoes. This means that in addition to the concepts of *reality* and its *understanding*, a third fundamental concept must mediate between them: the *perception* or *observation* of reality. This, then, is the tripod of the conceptual schema on which the SM must rest:

### Reality

There is a triple zero hypothesis prior to the establishment of scientific understanding: (1) reality exists; (2) a mind capable of understanding it also exists; and (3) a certain interaction between them is possible. In particular, the mind is capable of perceiving reality and of organizing successive perceptions in order to observe it. With these opening words, we have already named the three fundamental concepts of the schema we want to build: reality, observation, and comprehension. The necessary definitions are given below.

#### Slice of Reality R(Ω, τ)

Slice of reality* R*(*Ω*,* τ*) is a distribution of matter, energy, and information contained within a ***Ω*** region of space and a ***τ*** period of time.

#### Real Object RO

Real object RO is a slice of reality for a particular instant ***tЄτ*** being fixed, i.e., ***R(Ω, tЄτ)***.

#### Real Phenomenon RP

Real phenomenon RP is a slice of reality for ***r*** points of the space of a volume ***v*** *C* ***Ω*** being fixed, i.e., ***R(rЄv*** *C* ***Ω, τ)***.

The object of a piece of scientific knowledge always refers to a slice of reality. We will assume that the reality exists even when there is no observer in a position to perceive it. The perception of reality depends on the time and place. To the naked eye, perceivable reality is in fact very limited: the slice of reality may be imperceptible because it is too large or too small, too opaque or too transparent, too far away or too close, too quick or too slow, too complex, and so forth. Over the course of history the mind has managed to widen perceivable reality with the help of instruments that act as exosomatic extensions of the ability to see (telescopes, microscopes, high- and low-speed cameras, scanners that operate at different frequencies, etc.).

### Observation (of Reality)

To perceive reality implies a kind of conversation between a mind and a slice of reality. The mind devises a representation of a slice of reality by using some kind of language. When the perceptions are programmed in accordance with preconceived criteria, the perception is called observation. When the observation arises from imposing certain particular conditions, the observation is called experimentation. These are the definitions linked to this concept that we require.

#### Language L_m_

Language L_m_ is the collection of *m words* (letters, magnitudes, variables, notes, lines, symbols, etc.) that are combined to compose phrases (propositions, equations, images, sounds, etc.), and with them *texts* that are employed to represent a slice of reality.

#### A perception L_m_ R

A perception L_m_ R of a slice of reality ***R*** is a text of words and propositions in a language ***L***_***m***_ that represents a slice of reality ***R*** with a particular spatial resolution* ΔΩ* and a particular temporal resolution ***Δτ***.

#### The Spatial Resolution ΔΩ

The spatial resolution* ΔΩ* of a piece of knowledge is the size of the region of space in which the representation is (or is considered to be) invariant. The *temporal resolution* ***Δτ*** of a representation is the length of the period of time in which the representation is (or is considered to be) invariant. The *size* ***|L***_***m***_ ***R|*** *of a perception* ***L***_***m***_ ***R*** is the number of symbols (letters, words, propositions, etc.) used in it. The *universe of reference* ***U*** of a slice of reality ***R*** is a set of slices of reality ***R*** ^***j***^ constructed or selected bearing in mind the differences with ***R***: ***{R,R*** ^***j***^ ***}*** for *j* = *1*,*2*,…*n.*

#### An Observation O

An observation O of a slice of reality ***R*** is the set of representations in accordance with ***L***_***m***_ of all the *m* slices of reality of a universe of reference included in the slice *R* to be observed:

$$ O = \left\{ {L_{m} R,L_{m} R^{j} } \right\} \, j = 1,2, \ldots m. $$

#### The Size |O|

The size |O| of an observation is the number of words in ***L***_***m***_ of the representation.

Every observation of reality consists, then, of perceptions of reality. It is quite possible for a reality to be perceived but for it to be difficult or impossible to observe. For example, the elliptical trajectory of a planet around a star is perfectly observable if it is perceivable because we can break down the movement into spatial and temporal elements in order to ascertain the differences between similarities (a single planet around a single star, different planets around the same star, different planets around different stars, etc.). The behavior of a galaxy can be perceived in an instant, but its evolution over time is difficult to observe due to its slowness in relation to the time allotted to an observer. In this situation, however, it is always possible to construct a universe of reference using the perception of different galaxies of different ages in different conditions. In contrast, what we call a mystical experience can be perceived, but it is very difficult to observe. With these intuitions, we can already put forward two of the fundamental concepts of the schema we wish to build: reality and understanding (of reality).

The concept of observation can be summed up as a construction achieved by means of *differences between similar realities.*

### Comprehension (of the Observation of Reality)

Arriving at an understanding of reality is the central concept of the SM. Curiously, it admits of a definition symmetrical with the earlier concept of observation. If the observation is a construction built up of differences between similarities, then understanding can be defined as a construction achieved by *similarities between different realities*. As in the case of observation, understanding requires a language.

#### A Comprehension C

A comprehension C (of an observation ***O*** of a slice of reality ***R*** in relation to a universe of reference ***{R,R*** ^***j***^ ***}***) is a representation in accordance with ***L***_***m***_ of the possible intersections between the slice of reality ***R*** and the other slices of reality that make up the universe of reference ***R*** ^***j***^, that is to say, an understanding is a representation of similarities between differences.

That is, for a particular language **L**_**m**_, understanding can be represented as

$$ C = \{ R \cap R^{j} ,R \cap R^{i} \cap R^{j} ,R \cap R^{i} \cap R^{j} \cap R^{k} , \ldots \} \;for\quad i,j,k = 1,2, \ldots ,q $$

In other words, understanding is made up of everything shared in common by the various slices of reality of the prepared universe under observation.

Accurate observation of the motion of the planets, for example, leads to a differential equation that compresses all understanding of such a slice of reality. And from such an understanding, it is possible to anticipate and reconstruct the motion of any planet in any galaxy in the universe.

However, some things are not scientifically understandable. In the case of a mystical experience, scientific comprehension seems unattainable. The experience in itself is perceivable, yet planning an observation is practically impossible—how can one define a universe of observation with an unrepeatable slice of reality?

The degree of intelligibility of scientific understanding calls for a number of additional definitions: The *size* of an area of comprehension |***C|*** is the number of words in ***L***_***m***_ of the comprehension ***C***. The *universality* ***U*** of an area of comprehension ***C*** is the reunion of all the slices of reality that share this comprehension.

$$ U = \cup_{\upsilon } \{ L_{m} R^{\upsilon } |C \subset L_{m} R^{\upsilon } \} \quad\upsilon = 1,2, \ldots_{{}} $$

An observation and an understanding are always finite. Yet the domain of validity of an area of comprehension, its universality ***U***, may be infinitely large. This is not to say that two infinite universalities must necessarily be the same in size. For example, the laws of classical mechanics and those of relativistic mechanics are finite. However, both theories have infinite universalities even though classical mechanics will always be contained in relativistic mechanics when the reverse is not true. Cantor’s (1915) theory is clear in this respect. There are infinite natural numbers, infinite rational numbers, and infinite real numbers, yet the infinity of real numbers is greater than the infinity of rationals, and the infinity of rationals is larger than the infinity of naturals.

##### The Degree of Universality

It is possible to establish an order between different universalities, ***U1*** and ***U2***, simply by defining that the degree of universality of ***U1*** is greater than that of ***U2*** if the first contains the second. This criterion is fundamental if the SM is, as we propose, to demand the maximum universality possible.

##### The Degree of Intelligibility **μ**

The degree of intelligibility **μ** of an area of understanding depends on the relationship between the size of this understanding and the size of the observation that preceded it. There are two aspects to understanding: one derives from what is common to what is different, and the other derives from the simplest expression of the first. In this latter respect, the greater the compression, the greater the understanding, enabling us to formulate the degree of intelligibility in the following manner:

$$ \mu = 1 - \left| C \right|/\left| O \right| \, \mu \in \left[ {0,1} \right] $$

If ***μ*** **=** **1**, intelligibility is at its maximum and it occurs when maximum comprehension corresponds to maximum compression: ***|C|*** < < ***|O|***. At the other extreme, intelligibility is at its lowest when ***μ*** **=** **0**, i.e., when the observation is in itself also the best understanding: ***|C|*** = ***|O|***. Chaitin-Kolmogorov complexity theory illustrates this way of seeing things (see the “Selection of the Comprehension (OC) of a UO” section).

There are, then, *degrees of intelligibility*. The degree of intelligibility of planetary motion is high since the size of the understanding is finite (Newton’s three laws and the law of gravitation), whereas the size of the observation may be made infinitely large. Another slice of reality, such as the behavior of a family group of gorillas, will undoubtedly result in the size of the observation and the size of the understanding being much closer. Different ways of understanding a single reality can, therefore, be arranged in order according to their degree of intelligibility. Kepler’s laws, for example, are a good understanding of the motion of the planets around the sun, but Newton’s laws have a greater degree of universality and intelligibility. The degree of intelligibility will also lead to a good criterion if it is appropriate, as is the case, that the SM should demand the highest possible intelligibility.

## The Three Fundamental Hypotheses (the Limits of Scientific Understanding)

**Fig. 1:**
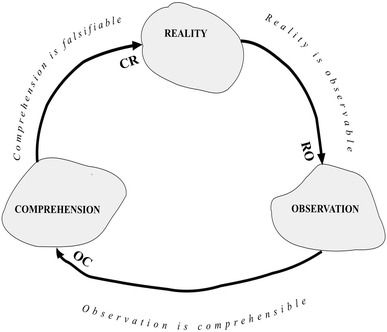
The three fundamental hypotheses

The SM that we are trying to design should be applied to observable realities, understandable observations, and understanding not shielded in advance against what may occur in reality. Figure 1 shows the connections between the three fundamental hypotheses: (1) reality is observable (RO); (2) observation is comprehensible (OC); and (3) comprehension is falsifiable (CR) in relation to the three initial concepts of reality (R), observation (O), and comprehension (C).

### Reality is Observable (RO)

Not all realities are necessarily observable, but science deals with those slices of reality that are. It is quite possible in the case of a particular reality for it to be impossible to build a universe of reference made up of other realities that are directly or indirectly comparable. This, for example, was the case with many elementary particles prior to the construction of particle accelerators, and it is also the case with many aspects of cosmology. The arguments of certain critics of superstring theory (Glashow and Ginsparg^1986^) are founded on the impossibility of observing or of managing to observe it at work.

The unbridgeable gap that prevents us from scientifically understanding a superstition lies in the impossibility of observing it at work. As will be seen, this hypothesis is essential for stating the *first principle of the scientific method* or the *principle of objectivity*.

### Observation is Comprehensible (OC)

The observation of any slice of reality is not necessarily understandable, but science deals with those observations that are. Einstein’s remark that “the most incomprehensible thing about the world is that it is at all comprehensible” is in this context pure irony. If we accept the definition of C, the most sensible thing is to hope to find coincidences between the differences when two realities are compared. Metaphorically speaking, we can say that two branches have something in common when they belong to the same tree. An incomprehensible reality would correspond in the metaphor to a forest with more trees than branches. And, in accordance with prevailing cosmology, all realities have at least one thing in common, their history. Intelligibility, however, also has another meaning related to the weight of its expression. In Chaitin and Kolmogorov’s algorithmic information theory (Chaitin 1966), to give another example, an incomprehensible observation is one generated by an algorithm no shorter in length than the sequence of digits that represents the observation itself. In this sense of being understandable by being compressible, the incomprehensible arises when the best understanding is directly the minimal observation. In the following section, we will discuss how these two meanings of intelligibility are combined.

An observation may be completely incomprehensible if the universe of reference has not been well selected. For example, we are unlikely to arrive at an understanding of the trajectory of a body launched in a field of constant gravity if the positions, instances, and speeds have been chosen from motions with arbitrary initial conditions.

The nonfulfillment of this hypothesis in either of its two senses will push intelligibility to its lowest degree. Science has nothing to contribute beyond this limit. As will be seen, this hypothesis is essential for stating the second principle of the scientific method, the *principle of intelligibility*.

### Comprehension is Falsifiable (CR)

Not every understanding is falsifiable in the Popperian sense of the term (Popper 1959), but science deals only with understandings liable to enter into direct or indirect contradiction with reality. In fact, falsifiability does not necessary involve systematic observation. To determine the non-falsifiability of a piece of presumed scientific understanding, all that is required is a simple perception of the reality or the possibility that such a perception may be imaginable by the individual seeking knowledge. This is the meaning of the direct relationship that can be established between the understanding and its reality in Fig. 1.

Falsifiability is necessary for paradoxes of contradiction to arise between reality and the understanding of it when both exist but it turns out that they are incompatible, or for paradoxes of incompleteness to arise when one exists without the other (i.e., when a reality exists without the corresponding understanding or an understanding exists without a corresponding reality). All those cases in which understanding is unaffected by everything that may occur in reality are outside the scope of science. For example, a prediction that covers every possibility is assured of being compatible with reality, yet it does not enter the realm of science because understanding can never enter into conflict with the understood. A belief is just a belief, and no more than a belief, if it is completely armored against anything happening in reality. That which encompasses everything understands nothing. Incoherence is the greatest means to avoid falsifiability, since if a proposition is not correct, then its negation will always be right. To attribute good luck to the satisfaction of the gods and ill fortune to their anger is a belief that cannot possibly be dealt with scientifically. As will be seen, this hypothesis is essential to the statement of the third principle of scientific method or the *dialectical principle*.

The three fundamental hypotheses, like every working hypothesis, are neither the truth nor lies: they are either accepted or they are not. In the case of scientific method, the three hypotheses (Fig. 1) constitute an overall set of criteria for demarking *the scientific*, in other words, the realm in which the scientific method can be applied.

## The Three Fundamental Principles (Scientific Method)

**Fig. 2:**
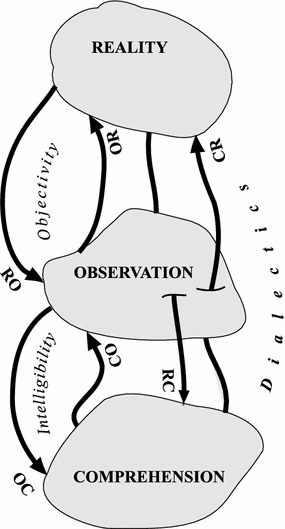
The three fundamental principles

The understanding of a slice of reality is scientific if the three principles stated below are abided by. Figure 2 shows the three principles of scientific method and their relationship with the three fundamental concepts (reality, observation, and comprehension).

### Principle of Objectivity: Observation is Maximally Objective

This principle affects the observation of a slice of reality and has two meanings. The first refers to the distortion that an observation may cause in the slice of reality being observed merely due to the process of observation itself (OR). The second refers to the opposite distortion, which a particular slice of reality may cause in the observation by the mere fact that its nuances may mask the essence (RO). The principle of objectivity is, then, a two-part recommendation to guide the selection of (1) the chosen method of observation, and (2) the slice of reality chosen to be observed. These two sub-principles establish a tendency to be followed as closely as possible by the scientific method, and are formulated in the following manner:

#### Selection of the Particular Manner of Observing a Particular Slice of Reality (OR)

Scientific method recommends that of all the available ways of perceiving a slice of reality, the one chosen should be the one that least distorts the observed. The direct benefit of this will be to attain the maximum *universality of the science vis*-*à*-*vis the observer*, in other words, the least influence from her particular beliefs, prejudices, or circumstances.

#### Selection of a Particular Universe of Observation Based on a Slice of Reality (RO)

For a particular a slice of reality, one arrives at a set of slices of reality called a universe of observation (UO) by means of differences established in accordance with well-defined parameters (such as time and/or space). An individual observation of each of these slices of reality is obtained, so the UO is a set of pairs in which each pair consists of a particular slice of reality and its corresponding observation, which, as mentioned above (in “Selection of the Particular Manner of Observing a Particular Slice of Reality (OR)”), is the observation that least distorts it. The direct benefit of this is to achieve the *universality of science vis*-*à*-*vis the observed*, in other words, that which determines the breadth of the field of validity of the resulting knowledge or, to put it another way, that which makes the difference between a fundamental law, a phenomenological law, or a simple ad hoc model.

Both the first hypothesis (the “Reality is Observable (RO)” section) and the first principle (the “Principle of Objectivity: Observation is Maximally Objective” section) may be grouped together under the term “hypothesis of the real world,” which is indebted to a reflection of Erwin Schrödinger (1944), who in turn drew his inspiration from ancient Greek philosophy.

### Principle of Intelligibility: The Understanding is Maximally Intelligible

This principle affects the understanding of an observation and governs a process that can go in one of two directions: firstly, from observation to comprehension (OC); and secondly, from comprehension to observation (CO).

#### Selection of the Observations (CO)

Selection of the Observations (CO) of a universe of observation (UO) that have the highest possible intersection, which we will term simply the *intelligibility* of the UO.

Example: A good universe of observation consists of all the movements that are not too fast of bodies that are not too small. What they have in common are the fundamental laws of classical mechanics.

#### Selection of the Comprehension (OC) 

Selection of the Comprehension (OC) of a UO. This is the most compact way of expressing intelligibility. *The most compact form of understanding is arrived at using Newton’s laws.* The dual idea (“Selection of the Observations (CO)” and “Selection of the Comprehension (OC) of a UO” sections) can be summed up by saying: the principle of intelligibility tends to determine the minimum of a maximum. The maximum emerges directly from the sense of the concept according to the “Selection of the Observations (CO)” section (understanding is the maximum in common) and the minimum proceeds from the “Selection of the Comprehension (OC) of a UO” section, inspired by the old idea of Ockham’s razor (when two explanations give an account of equal merit of a slice of reality, the simplest is chosen). The combination of these two ideas driving in the opposite direction has been explicitly stated by various authors, and tacitly suggested by many others. In effect, the two senses of understanding (as *the common between the diverse* on the one hand, and comprehension as *compression* on the other) are not alternatives, nor are they contradictory. Philip Kitcher (1981), for example, comments on this seeming dilemma when he stresses the contrast between the strong intuition expressed in the phrase *understanding is to do with the idea of reducing unfamiliar phenomena to familiar phenomena* (the idea of reduction) and Hempel’s intuition (the idea of the common), expressed as follows:

What scientific explanation, especially theoretical explanation, aims at is not (an) intuitive and highly subjective kind of understanding, but an objective kind of insight that is achieved by a systematic unification, by exhibiting phenomena as manifestations of common, underlying structures and processes that conform to specific testable, basic principles. (Hempel 1966, p. 83)

Feigl (1970, p. 12) also sums up the integration of these two selfsame meanings with equal priority: “The aim of the scientific explanation throughout the ages has been ‘unification,’ i.e., the comprehending of a maximum of facts and regularities in terms of a minimum of theoretical concepts and assumptions.”

Both senses have their tradition in history, albeit separately. The first meaning (understanding through what is common to the diverse) is known in the literature on the subject as “understanding as unification” and has been well argued by authors such as Weber (1996). The second meaning (comprehension through compression) is brilliantly defined in Chaitin and Kolmogorov’s algorithmic information theory (Chaitin 1966), which defines the complexity of a sequence of data as that of the shortest algorithm that generates it. The more compressible the data of an observation, the greater the degree of understanding. A sequence of a million figures of the type **010101010101**… is highly compressible (and hence highly understandable) to the much shorter proposition, for example, of **PRINT0110EXP6TIMES**. In contrast, the results of the last million football games played around the world generate a totally incomprehensible series of digits. The best way to represent these data is the sequence of data itself. Consequently, we find ourselves at the opposite extreme of the degree of intelligibility: it is the limit of zero compression and hence also of the zero degree of comprehension. Consequently, there are, as in the case of objectivity, also degrees of understanding, which lie somewhere between a maximum and a minimum.

With this principle, scientific understanding acquires a clear and profound utility, which is nothing less than the ability to anticipate in the broadest meaning of the term. We will discuss this in more detail below.

The second hypothesis and the second principle of the SM are indebted to two intellectuals: (again) Erwin Schrödinger (1944), and the medieval thinker William of Ockham (Hempel 1966).

### Dialectical Principle: Understanding is Coherent (Without Paradoxes of Contradiction) and Complete (Without Paradoxes of Incompleteness)

This principle ensures that the validity of an area of understanding remains up-to-date due to its sensitivity towards the same reality. It establishes that scientific comprehension tends to be maximally coherent and complete. Between the slice of reality understood and the slice of reality perceived (or observed), two types of paradoxes may arise: paradoxes of contradiction, and paradoxes of incompleteness. Both cases are resolved by new comprehension (RC) or by a new reality or way of perceiving it (CR). In both cases, we can talk of the advancement of scientific understanding.

#### A Paradox of Contradiction

A paradox of contradiction arises when an incompatibility occurs between the understanding of a slice of reality and its perception. In this case, there are two options for restoring coherence: to change the understanding or to change the slice of reality (or the perception of it).

##### New Comprehension Through a Paradox of Contradiction (NCPC)

If the incoherence is overcome by new comprehension (RC), then what we have is a scientific revolution (the new understanding replaces the obsolete understanding, the validity of which is at an end). All new understanding can be termed a revolution.

Example: On 4 June 1999, a well-known generalist science magazine published an article that put an end to more than 100 years of contradiction between the then-prevailing theory and an observation of reality. The authors included the theoretical physicist Geoffrey B. West and the biologists James H. Brown and Brian J. Enquist. The irreconcilable difference was between the value of the allometric exponent, which relates an individual’s mass with its basal metabolic rate, observed in an extremely broad universe of observation in reality (in the animal kingdom, from a tiny shrew to a huge whale), and the value deduced from the most reasonable hypothesis compatible with prevailing thermodynamics. The first value was 3/4 and the second was 2/3; the first is “what we saw” and the second is “what we believed” on the grounds that the energy produced in a volume (proportional to the cube of the distance) should dissipate through the surface that separates it from the outside (proportional to the square of the distance), assuming that the heat is generated, as in a stove, uniformly at every point of the interior. The authors of the article modified the hypothesis by assuming that heat is not generated uniformly in the body but above all in the fractal structure of the circulatory system. With this new hypothesis, a new understanding was arrived at and a total coincidence on the constant of 3/4 was achieved.

##### A New Reality through a Paradox of Contradiction (NRPC)

If, on the other hand, the paradox of contradiction is resolved by changing the slice of reality affected by the contradiction, then what we have is the emergence of a new slice of reality (CR). This is when the domain of validity of the understanding alters (for example, it shrinks) or when we gain a better perception of this slice of reality. In this case, we cannot speak of a scientific revolution but we can perhaps talk of scientific evolution or progress. What is achieved is a new reality without paradoxes with the prevailing theory.

Example: In November 2011, CERN reported that it might have detected neutrinos traveling faster than light. The contradiction with the special theory of relativity had the scientific community in an uproar (Wright 2011) for several weeks. However, a technical error was soon discovered, resolving the matter in favor of the prevailing special theory of relativity, which was thus strengthened.

#### A Paradox of Incompleteness

A Paradox of Incompleteness arises when a non-understood reality or an understanding not perceived in the reality is detected during an observation of reality, in other words, when there is a lack of understanding or when there is a lack of reality. This paradox has two forms, then.

##### New Comprehension Through a Paradox of Incompleteness (NCPI)

The first is illustrated by the phrase “I don’t understand what I perceive” and is resolved by a scientific revolution (RC).

Example: Over the millennia, numerous eyewitnesses have been amazed when they have had the unlikely opportunity to see the spontaneous and capricious evolutions of certain fireballs during dry storms in the desert. Dozens of magical and mysterious interpretations have been put forward since antiquity. Various thinkers such as Seneca, Benjamin Franklin, Nikola Tesla, and Niels Bohr have attempted to use intelligible knowledge to provide an explanation that would make it possible to reproduce these spheres of fire (which vary in diameter from one centimeter to a meter). Since then, a number of groups of researchers have tried to simulate or reproduce the phenomenon in the laboratory. Eventually, Professor Pavão and his collegues (2007) managed to reproduce these mysterious fireballs and their behavior in the laboratory. The word “mystery” is the name for failed understanding of the “I do not understand what I can see” type; in this instance, it took several centuries before a coherent understanding of the phenomenon was arrived at.

##### A New Perception of Reality Through a Paradox of Incompleteness

The second form of the paradox of incompleteness is illustrated by the phrase “I cannot perceive what I understand” and is resolved by improving the observation (CR).

Example: On 4 July 2012, CERN announced that it had in all likelihood detected the so-called Higgs boson. For more than 40 years, the Standard Model of elementary particles had predicted the existence of a particle that no one had been able to see (Witze 2012). During all this time, there had been a paradox of incompleteness of the “I cannot see what I understand” type.

The falsifiability of an area of understanding can be directly established with the reality, or indirectly with the mental representation of a reality. A non-falsifiable understanding is compatible with any kind of reality, regardless of whether it has been observed or not. It is shielded against anything that might occur in reality. One can also speak here of degrees of falsifiability. The highest degree of falsifiability arises when it is possible to design an experiment whose results may contradict understanding, and the lowest when the understanding is logically shielded against reality (for example, “tomorrow one of two things will happen: either there will be a solar eclipse or there will not”). This suggests that the third principle of SM is simply the formula with the highest degree of falsifiability available.

The third hypothesis and the third principle of SM are clearly indebted to Popper (1959) and his demand that understanding should not shield itself from reality, to Hegel’s dialectic (1977) on the cognitive power of contradictions, and to the even more ancient roots of Plato’s ideas.

## The Three Fundamental Benefits of the Scientific Method (The Nature of Scientific Comprehension)

**Fig. 3:**
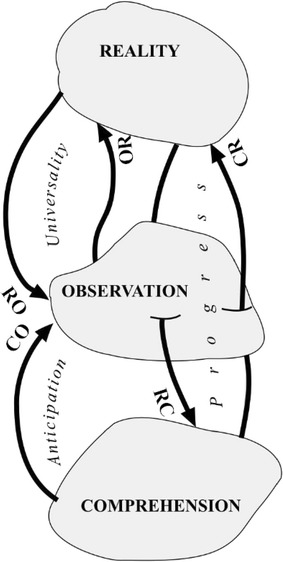
The three fundamental benefits of the scientific method

For each of the three hypotheses of the scientific method there is, as we have seen, one of the three fundamental principles. And now we will see how a great benefit of knowledge is obtained from each of these principles. We have already noted what the properties are that SM confers on science. From objectivity between reality and observation we derive *universality* vis-à-vis both the observed and the observer; from intelligibility we derive *anticipation* vis-à-vis the understanding of observation; and from the dialectic between understanding and reality, we derive the capacity for *progress* of science, both in the sense of arriving at new understanding and in the sense of promoting new realities (see the following sections). Any other form of knowledge, such as artistic or revealed knowledge, may be universal and it may display one or more of these virtues, but it is scientific knowledge that always ensures the highest degree possible of these virtues at all times and in every place. And this it does through the construction of the SM. Figure 3 shows these virtues in the triple supporting schema of reality, observation, and understanding. We will now go on to discuss these relations in detail.

### Universality

Universality is achieved in science in two ways (OR and RO) thanks to the principle of objectivity that governs the relationship between a slice of reality and its universe of observation.

#### Universality vis-à-vis the Observed (RO)

In the direction that goes from reality to observation, the creation of a universe of observation, consisting of the maximum intersection of slices of reality possible, ensures that the scientific knowledge obtained is as independent as possible from that which is observed. In other words, this sense of the principle of objectivity tends to ensure that the domain of validity of the reality to which it is applied is as large as possible. This is what distinguishes a big theory such as quantum physics from a phenomenological law such as Ohm’s law, and this in turn from an ad hoc model such as Lotka and Volterra’s law on the interaction between predators and prey.

#### Universality vis-à-vis the Observer (OR)

In the opposite direction, i.e., from observation to reality, the principle of objectivity requires that the observation should alter the observed as little as possible, as a consequence of which another type of universality is ensured. In this case, the impact of the particular preconceived ideology of the observer, or her particular way of observing, are tempered. Clearly, the more complex the object, the more complicated this independence will be. The observer will influence the trajectory of a billiard ball less by watching it than he will the behavior of an animal or while interviewing a patient on a couch. However, the SM offers tendencies and even though the objectivity of an economic observer will never attain the level of objectivity of an astronomer, both deserve equally to be described as scientific if they both apply their objectivity to the full.

### Anticipation

This is the ultimate goal of the first utility of science. Understanding reality in the sense of the principle of objectivity of SM makes it possible to anticipate uncertainty, an essential faculty for the survival of any being that lives in the real world. Anticipation is a term that here acquires a broad meaning. In fact, it not only means to anticipate in time that we cannot as yet see because it has not as yet occurred (an eclipse anticipated centuries beforehand), but also means to “anticipate” in time that which has already occurred but for which there was no possibility of direct observation due to a lack of observers or the means to do so (the geology of a landscape millennia later). It also means, however, to “anticipate” in space in the sense that scientific knowledge allows us to speculate on phenomena that we cannot see because there are barriers in space that block this observation (other planets, other galaxies, etc.). In this case, it is perhaps more appropriate to extend the meaning of the term “anticipate” than to invent a neologism. Here, then, is the true meaning of understanding that I advocate: the understanding of reality anticipates in the sense that understanding serves to replace the act of observing itself (CO). One observes in order to understand, but with the prevailing understanding at hand one no longer needs to observe everything. Understanding replaces difficult, awkward, or impossible observations. This is its profound meaning. What purpose does understanding serve? The answer could not be weightier: it is undoubtedly the best strategy for surviving in the face of uncertainty. We have arrived by natural selection at cultural selection, so scientific understanding is an achievement with a great evolutionary tradition.

### Progress

Science progresses thanks to, among other things, the dialectical principle that governs the relationship between reality and our scientific understanding of it. As we have seen, there may be two types of progress: the generation of new understanding, or the generation of a new reality. The first means that preexisting knowledge becomes obsolete when it is overtaken by the new; the second indicates that there is an error in the perception of reality (as in the aforementioned case of the detection of particles travelling faster than light), or that the universality of the prevailing knowledge now has a smaller domain of applicability (the prevailing knowledge is more limited or more confined: the theory of relativity does not rule out Newtonian mechanics, but restricts it to low-speed scenarios). There is little to add, then, to the discussion in the section above discussing the “Dialectical Principle”. The advance of science is possible in accordance with the alternatives described:

#### Progress Through New Understanding (RC)

In this case, the progress of science consists of the generation of new understanding, which in turn may occur, as we have seen, in two ways: via the paradox of contradiction, or via the paradox of incompleteness.

#### Progress Through New Reality (CR)

In this case, the progress of science consists of suggesting changes to the way reality is perceived, which, as we have also seen, may take place through the paradox of contradiction or the paradox of incompleteness.

This does not exclude the possibility that other intuitions, even those from outside science, may initiate a process of renewal of scientific knowledge without the need for coming across any kind of paradox with reality. Indeed, SM is not useful for acquiring ideas that will lead to renewal but it is for dealing with them.

## The Three Intellectual Joys (Psychology of the Acquisition of New Scientific Knowledge: Research and Education)

The scientific understanding of reality is an activity of the mind well equipped for survival. Gaining scientific understanding became, in evolutionary terms, a vital function for *Homo sapiens*. Now, a vital function is always essential for the individual and his genes to endure: hunger ensures feeding, thirst hydration, pain care of one’s own health, sexual attraction descendents, and so on. Natural selection favors the consolidation of stimuli without which all these vital functions could be postponed—fatally postponed. Natural selection helps to overcome the obstinate tendency of every living being to spend the minimum energy and to expose itself as little as possible to uncertainty. This is something that could easily be termed the universal principle of the laziness of matter: faced with the dilemma of choosing between *doing* and *not doing*, the individual tends to incline from the start towards *not doing*. This is the function of stimulus: to avoid long postponements when urgent needs exist. Understanding, and especially scientific understanding, is a vital function that has very recently appeared in evolution, and it is more than likely that natural selection has not yet had sufficient time to establish certain innate and indomitable stimuli to its benefit. However, it is possible to speak of a certain intellectual gratification that operates in the manner of cultural stimulation. This is what we might appropriately call intellectual joys, of which there are three broad groups. The most surprising aspect is that each type of intellectual joy is closely associated with one of the three principles of the proposed SM.

There is an intellectual joy associated with the principle of objectivity that we will term intellectual joy through conversation; another associated with the principle of intelligibility, intellectual joy through understanding; and a third associated with the dialectical principle, intellectual joy through paradox.

In short, SM is designed to guide the creation of new science (research), but it is also useful for guiding the passing on of science (education). Every process aimed at the acquisition of new knowledge–whether “new” refers to a particular citizen (education) or all the citizens in history (research)–can be sensibly divided into three phases: (1) stimuli, (2) conversation, and (3) understanding. Conversation is prompted by the stimuli, and understanding is prompted by some kind of conversation. The connection between these three ideas and the three principles of SM is described below.

### Intellectual Joy Through Paradox

The process that leads to the acquisition of new scientific knowledge begins with a stimulus. In which situations is intellectual joy through paradox generated? I believe that this can be precisely determined. The key lies in the third principle of scientific method, i.e., in the dialectical principle. It occurs when the creating mind perceives a threat to a prevailing piece of knowledge. At that very moment, the mind of the scientific subject experiences what we might call intellectual joy through paradox. As described above in the section on the “Dialectical Principle,” this can occur with a paradox of contradiction or a paradox of incompleteness. The mind intuits that it must fight the environmental uncertainty in order to survive (let us not forget that this intuition inspires this principle of SM). From this, two pedagogical recommendations emerge: the first refers to how good paradoxes should be sought, the second to the best way to use them.

#### The Educational Value of Paradoxes: Looking for and Using Paradoxes

Almost everything in our educational systems is based on representations of reality: the teacher’s discourse, books, videos, computers, etc. What is lacking for the occurrence of paradoxes is delving into reality itself. The tendency to hide paradoxes, as usually happens in many teaching institutions, is a gross error. A good teacher, in contrast, does not evade contradictions but looks for them. This means that a considerable proportion of teaching needs to be programmed outside the classroom; in other words, time needs to be invested in going out into the outdoor reality to gather stimuli. There is nothing more stimulating than reality itself. Consequently, why not “Reality” as a school or university subject? It is the best way to stir intellectual joy through paradox.

The educational system tends to present science as a closed doctrine to which the pupil comes lamentably too late to contribute. Showing the method and discussing errors is educationally very valuable in this early phase in which the mind passes from a state of indolence to a state of keen interest in learning. For example, science museums tend to show the results of science but not the path that led to them. The message of a rounded, perfect science, complete and without cracks, looks nothing like a good stimulus. There is no better conversation starter than a good paradox.

### Intellectual Joy Through Conversation

The conversation begins when the individual in search of knowledge has received sufficient, adequate stimuli. Conversation is not, however, valued in the classroom where, in general, the discourse tends to flow in just one direction, from teacher to pupil.

When and in what conditions does intellectual joy associated with conversation occur? There is a subtle answer to this subtle question. Any form of conversation on SM consists of an exchange of questions and answers between two interlocutors, one of whom is always the human mind (the subject–in other words, the individual wanting knowledge) while the other may be the perceived or observed world (the object of knowledge), any other mind (the exchange of ideas) or the subject’s own mind (reflection). Intellectual joy associated with conversation occurs whenever conversation supplies some kind of innovation, that is to say, when the conversation does not shut itself in by returning to the starting point but opens up and takes different directions, so the point of arrival does not coincide precisely with the point of departure. It is when the perfect (vicious) circle turns into a kind of (virtuous) cycloid. The precise moment of intellectual joy through conversation occurs at the exact instant when the mind grasps that a point of arrival does not coincide with any point visited before.

This intellectual joy is directly related to the first principle of SM, with the principle of objectivity, which governs the best conditions in the interaction between the subject and object. As indicated below, recommendations of extreme usefulness to education can be drawn from this.

#### The Educational Value of Conversation

The second phase of the cognitive process is centered on conversation, which is present in every process of new scientific knowledge acquisition, and good ideas for education can be drawn from this as well. Conversation is not difficult to define and is framed in the alternation of the reception and transmission of ideas: *listening before speaking*, *speaking after listening*.

Everything in science is imbued with some kind of conversation, but neither can there be teaching or education without conversation. The educational system must therefore place value on conversation and train pupils in its art, and it should be treated as an intellectually healthy and useful activity. This conclusion is of course not news, but it is something we have forgotten. It is the famous peripatetic method employed by Aristotle, in which master and students converse as they walk. Any educational system that does not allocate time and space to conversation contains a fundamental error in its core, as understanding is always produced at the end of any form of conversation.

The educational system today generally extends over some 20 years from nursery to a bachelor’s degree. As one advances from the start to the end of this period, one can easily see that the conversation between teachers and students becomes increasingly difficult and one-way. In classes crammed with students, all they can possibly do is listen. There are a wide range of subjects nowadays, so why isn’t there one given over expressly to conversation?

Everything to do with education should be conceived from the perspective of stimulating and fostering conversation, from the design of classrooms, lecture halls, and cafeterias, to the design of museums and the formats of the most diverse activities. For example, a museum, in which everything is up against the wall, as is usually the case, limits the likelihood of eyes meeting, thereby giving rise to a conversation. The design of gardens, cafeterias, and other meeting places in general ought to encourage conversation rather than inhibit it.

### Intellectual Joy Through Comprehension

We come at last to what we might call the moment of truth, the intense emotion generated by the acquisition of new understanding. Perhaps it could be said that new understanding always comes suddenly, like that “Eureka!” moment experienced by Archimedes. What are the conditions in which such an emotion is felt?

The clue lies once again in the SM, in its second principle, the principle of intelligibility, and more particularly it lies at the root of what it means to understand in science, as detailed in the “Principle of Intelligibility: The Understanding is Maximally Intelligible” section. These are the two columns on which scientific understanding rests: finding the maximum in common between different slices of reality (“Selection of the Observations (CO)” section) and reducing the expression of this to its simplest form (“Selection of the Comprehension (OC) of a UO” section).

#### Understanding Through the Maximum in Common

This point underpins much of the psychology of understanding. The mind constructs a universe of observation on the basis of a shared reality and is moved when it discovers, as it reviews them, additional intersections in principle unforeseen whose validity extends beyond the initial universe of observation. Common to the motion of the planets in the solar system is the fact that the planets orbit around a single star. However, it is finding first Kepler’s laws and then Newton’s laws as the element common to all these motions that produces the tremendous emotion of understanding. At the start comes the initial intuition, at the end the final (currently prevailing) understanding. The second confirms the first at the very moment of intellectual joy through understanding. The emotion of this understanding lies not only in the discovery of what is common hidden among various and different slices of reality, but also in the finding or suspicion that there are many more slices of reality that share the same comprehension. In addition, the validity of the understanding does not encompass just the planets observed but all the planets around a single sun, the planets of every sun, the suns themselves, and any body that travels through the gravitational fields of the cosmos. Understanding replaces observation. Archimedes’ legendary “Eureka!” sprang from a sudden understanding: the water that spilled from his bath, which measured the volume of his body, could also be used to measure the volume of any other irregularly shaped object, such as the king’s gold crown. The greater the number of slices of reality sharing an understanding, the greater the universality of that understanding and its corresponding psychological reward.

#### Understanding Through the Minimum of the Maximum in Common

Whereas the situation described above has to do with a maximum, this second situation has to do with a minimum. It is a process of reduction that consists of separating the essence from the nuances, the information from the noise, the central from the superfluous. This is understanding through compression. Every time the subject manages to reduce the essence of an area of understanding, intellectual joy occurs. This is another psychological gratification directly related to understanding. Polishing an area of understanding until it is completely free of any superfluous roughness brings with it, then, an intellectual joy. This also occurs in the leap from Kepler’s laws to Newton’s laws, mentioned earlier. This is the kind of intellectual joy that occurs when, for example, a language is replaced by another, more powerful one. Mechanics according to the cumbersome fluxions of Newton in his *Principia* is drastically reduced in the formulation of Hamilton or Lagrange in accordance with the language of infinitesimal calculus and differential equations. Landau’s and Lifshitz text (1960) developing rational mechanics on the basis of a variational principle is perhaps the expression of the greatest and most elegant synthesis of this discipline in physics.

#### The Educational Value of Understanding

All good education should encourage the occasioning of direct intellectual joy while ensuring that it is not short-circuited by any type of substitute. The ideal process is for the mind to look for and discover this joy for itself, guided by some form of conversation. The system of tests and exams to evaluate pupils usually becomes a kind of request for them to “admit” or “pretend” that they have learned. But believing the understanding of someone else is not the same as attaining it for oneself. The difference lies precisely in the occurrence or non-occurrence of intellectual joy.

How can the occasioning of true intellectual joy in the classroom be encouraged? There is no easy answer to this question, but it all revolves around creating the right conditions by offering for consideration by the pupil slices of reality linked together by the same *understanding* that is open in turn to undergoing later *compressions*. I would like to illustrate this point by mentioning an example taken from my own experience in modern scientific museography.

**Fig. 4:**
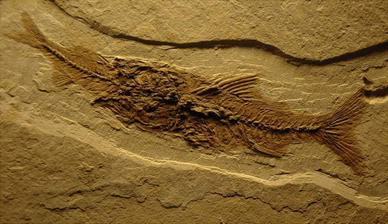
Photograph of a fossil on display at CosmoCaixa science museum in Barcelona. It is one of three fossils there all showing the same type of remarkable scene: the big fish is too small to swallow a small fish that is too big

On display in CosmoCaixa, the science museum in Barcelona, are three fossils of fish that share a single detail: in all three cases, one sees a large fish that has half swallowed a smaller fish (Solsona and Wagensberg 2002). The scene is remarkable—how is it possible that in the three cases the process of fossilization should have begun, tens of millions of years ago, right at the moment when one fish had half eaten another (see Fig. 4)? The mere contemplation of these three objects suggests that understanding is lacking. In effect, observing in reality a frequent phenomenon that the mind believes to be infrequent simply means that the mind lacks a certain understanding of reality. We have before us a paradox of incompleteness (as discussed in that-named section above). The universe of observation in this case consists of the three fossils that, though different, have something in common, which is precisely what has determined their selection. In this example, a fine intervention from the good teacher who wants to induce understanding perhaps consists solely in reminding pupils of the meaning of the concept of understanding in science and of encouraging them to find what other things might also be shared by such slices of reality (the maximum in common). Observation guided in this manner immediately bears fruit because in the three cases *the big fish is too small to eat a small fish that is too big*. In this way, intellectual joy suddenly hits the pupil (or the researcher in taphonomy studying the case for the first time). As a result, the first recommendation of looking for and finding the maximum in common is fulfilled. There is a second recommendation left, which is that the expression of this maximum should be minimal. For example, the large fish choked and the small fish drowned, causing the death of both, after which they were fossilized. The researcher (or teacher) then decides on whether it is appropriate to explore the validity of this theory by looking for more pieces in order to extend the universe of observation. This manner of inducing intellectual joy through understanding is already being successfully used in modern scientific museography, and in raising awareness of science (Wagensberg et al. 1996, 1997). However, there are serious doubts as to whether this idea can be transposed to schools and universities.

## Conclusions

There are two types of conclusions. The first is related to the existence, uniqueness, and validity of the SM (even outside science), and the second to the psychological implications of the SM, which are of particular importance in *research* and *education* programs.

### On the Existence and Uniqueness of the Scientific Method

The question of the existence and uniqueness of a scientific method can be resolved in a circular manner by proposing that we define science as any kind of knowledge produced in accordance with the three fundamental principles of the SM (as discussed above). There is, however, still one thing left to do: to match this definition with scientists’ perception of this form of knowledge over the course of history. Finding a discipline whose scientific nature deserves the consensus of the scientific community but which does not meet the three fundamental principles of the scientific method would be sufficient, for example, to cast doubt on both its validity and its uniqueness.

The key to the SM we present here lies in the fact that the three properties resulting from its application (objectivity, intelligibility, and the dialectic with reality) are not absolute values but admit degrees between a maximum and a minimum. This means that a particular understanding of a slice of reality may have a higher degree of objectivity than another rival understanding of the same slice of reality. The same may also be said of the degree of intelligibility or the degree of falsifiability. Now, given that the fundamental principles recommend the highest possible degree attainable in each instance, every piece of knowledge will be characterized in principle by a certain degree of “scientificity,” that is to say, the degree of intensity with which it was possible to apply the SM. This degree, which is influenced by the complexity of the slice of reality to be understood, is clear: the scientific understanding of the trajectory of a billiard ball will score more highly than the understanding of the behavior of a family group of mammals. But it should be said that, in accordance with the definition, both pieces of knowledge will be equally scientific if they both display the maximum degree available.

The SM simply recommends that objectivity, intelligibility, and the dialectical capacity of the knowledge be ensured to the maximum extent. This is how scientific knowledge of a particular slice of matter advances. Cosmology according to Ptolemy was science until Copernicus, and his was science until Kepler, whose theories were regarded as science until Newton, and, in all truth, Newton’s was science until Einstein. The SM also defines the validity of a particular piece of scientific knowledge.

Each of the three fundamental principles of the SM is necessary, but only the set of all three is sufficient. Fulfillment of the dialectical principle, for example, is a necessary but insufficient criterion. Astrology would score highly with the dialectical principle because there are no ambiguities or doubts concerning its falsifiability. However, it would fail catastrophically as regards its intelligibility and objectivity. Homeopathy, another example, may have few problems with its objectivity but it does in relation to its falsifiability (always hidden behind the placebo effect) and intelligibility. Psychoanalysis has often been criticized for its problems of falsifiability, yet the greatest or least consideration of the SM will produce more or less scientific versions of this extremely complex discipline. For good reason there are psychoanalysts who regard themselves as scientists and others who do not.

The task of reviewing the whole of the history of science armed with the SM far exceeds the ambition of this article, but suggesting it is part of its conclusions. The history of physics clearly emerges well from an analysis of scientificity using the SM, except perhaps for a few cases that are still the subject of fierce debate due to problems with their observation. This is unquestionably the case of superstrings or certain theories on complex systems. There can be no doubt that in these instances, the SM can be a good tool for debate and critique and for guiding those who find themselves immersed in these areas of research. The finding that the SM has been applied *avant la lettre* throughout the history of science is the equivalent of confirming that the scientist uses the SM, even if tacitly, whenever he engages in science.

In any event, there is another way of assessing the compatibility of the SM with what has been regarded over the course of history as scientific theories. This involves analyzing the various scientific methodologies on which these theories were based, and verifying whether they share what we have here put forward as an SM. A good historical and critical analysis of the science done via inductivism, via the conventionalism of Whewell and Duhem (1906), be it in honor of Popper’s views on falsifiability, Lakatos’s research programs, or even the examples raised by Feyerabend (1974) to deny the existence of a presumed scientific method, would confirm that the SM is a necessary and sufficient condition in every case (Lakatos 1971). None of these methodologies on its own is both necessary and sufficient. Popper’s idea of falsifiability, for example, has been the subject of lively debate, but in general this has been in the context of an overall philosophy of science. Falsifiability is, of course, necessary but it is not enough. In our suggestion, Popper’s sublime idea is contained in what we have termed the dialectical principle. Falsifiability in the SM does not represent an overall ideology but a powerful idea that serves to focus the demarcation of scientific knowledge and guarantees the possibility that scientific knowledge will advance. The SM is useful in order to do science but does not itself necessarily have to abide by its own principles. Something similar could be argued with regard to inductivism and the meaning that we here accord to understanding. There are two ingredients to our proposal concerning the notion of understanding in science: one is related to what is shared in common by things that are diverse; and the other is related to the minimal expression of what is shared. The idea of induction is based solely on the first of these two aspects.

The SM is thus a requisite to be fulfilled by any discipline that hopes to be described as scientific, but perhaps it is also something more, though not an obligation but an option. An artist, for example, can choose to be more or less scientific without this diminishing or adding to the merit of his work. A scientist does not have this freedom. A scientist can only be scientific if she employs objectivity, intelligibility, and dialectics to the fullest. Newtonian physics and psychology, for example, would be equally scientific because they strive to be as objective as possible. If the method is employed to the full, then psychology is scientific, but this is not true if the method is abandoned.

The SM is applied with decreasing intensity in accordance with the complexity of the slice of reality to be understood. Perhaps it is not too frivolous to arrange disciplines in decreasing order of how far it is possible to go with the SM: physics, biology, ethology, economics, sociology, and so on. The degrees of objectivity, intelligibility, and dialectics become increasingly difficult to attain as the complexity of the reality rises. We stress, however, that using the SM is a decision of the knowledge creator, and that the idea of obtaining results with the minimum preconceived ideology possible seems at the outset useful in all these disciplines. And it can be said that they are all equally scientific because in all of them the SM attempts to go as far as possible.

In contrast, one can be a great artist without the need to involve oneself with the SM. An artist can but he is not obliged to adopt the principle of objectivity: Albrecht Dürer and Alfred Hitchcock are two examples of artists who were not overly interested in distancing themselves from their work. An artist can but is not obliged to look for the simplest expression of the maximum that is shared in common. This was the choice made by Jorge Luis Borges, Pablo Picasso, or Salvador Dalí, but not by Vincent Van Gogh or Marcel Proust and, once again, they are no less artists because of that. The artist can play with paradoxes but is under no obligation to resolve them. Many artists go through a clearly scientific phase during which they look for their own language, but then move on from this stage once they have found it, as is the case with Antoni Tàpies and Joan Miró. Others explore new languages until the very end of their careers, as is true of Pablo Picasso and Antoni Gaudí.

### Education and the Scientific Method

Each of the three phases for acquiring new scientific knowledge is directly linked to one of the fundamental principles of the method: the stimulus phase to the dialectical principle; the conversation phase to the principle of objectivity; and the understanding phase to the principle of intelligibility.

#### Stimuli

Stimuli in general are a natural requirement (the product of natural selection) that guarantee the continuation of the vital functions of a living individual. Perhaps the closest we have to it are curiosity and play, which is a neotenic property in the case of humans. Pedagogically, the message is clear: immersion in the reality of the world is to be fostered. Or to put it another way, remoteness from reality is to be avoided. This brings us to the following phase, conversation. It is not surprising that the same principle of the SM that guarantees the advancement of science should also suggest good conditions for learning.

#### Conversation

Conversation is, in any of its forms, the essential path that leads to an understanding of reality. The first principle of the SM governs the way we should conduct those conversations that foster the twofold universality of scientific knowledge: independence from the particular reality to be known (the universality of the object) and independence from the particular ideology of the person who has the knowledge (universality of the subject). The importance of this when it comes to passing scientific knowledge on to others is beyond doubt. Good schools and universities and the most creative periods in the history of humankind (such as Florence during the Renaissance and Austria in the 1920s) make time and space for conversation.

#### Understanding

Understanding marks the moment of truth in education, and the second principle of the SM is devoted to it. If there is anything capable of instilling an addiction to knowledge it is the joy associated with understanding. Any hijacking, simulation, or substitute for intellectual joy is a serious handicap. The nub of the matter is that the last phase of the conversation that leads the pupil to attain understanding should be based above all on conversation with himself, reflection. It is not difficult to achieve this in museums. In classrooms, however, attaining this proves not to be so immediate, and it requires special research and effort.
